# Genomic Landscapes of EBV-Associated Nasopharyngeal Carcinoma vs. HPV-Associated Head and Neck Cancer

**DOI:** 10.3390/cancers10070210

**Published:** 2018-06-21

**Authors:** Hoi-Lam Ngan, Lan Wang, Kwok-Wai Lo, Vivian Wai Yan Lui

**Affiliations:** 1 School of Biomedical Sciences, Faculty of Medicine, The Chinese University of Hong Kong, Hong Kong 999077, China; lamjasonngan@gmail.com (H.-L.N.); 1155100020@link.cuhk.edu.hk (L.W.); 2 Department of Anatomical and cellular Pathology, Faculty of Medicine, The Chinese University of Hong Kong, Hong Kong 999077, China; kwlo@cuhk.edu.hk

**Keywords:** EBV(+) NPC, HPV(+) HNSCC, genomic profiles

## Abstract

Epstein-Barr virus-positive nasopharyngeal carcinoma (EBV(+) NPC), and human papillomavirus-positive head and neck squamous cell carcinoma (HPV(+) HNSCC) are two distinct types of aggressive head and neck cancers with early age onsets. Their recently identified genomic landscapes by whole-exome sequencing (WES) clearly reveal critical roles of: (1) inflammation via NF-kB activation, (2) survival via PI3K aberrations, and perhaps (3) immune evasion via MHC loss in these cancers as summarized in this review. Immediate outcomes of these WES studies include the identification of potential prognostic biomarkers, and druggable events for these cancers. The impact of these genomic findings on the development of precision medicine and immunotherapies will be discussed. For both of these cancers, the main lethality comes from metastases and disease recurrences which may represent therapy resistance. Thus, potential curing of these cancers still relies on future identification of key genomic drivers and likely druggable events in recurrent and metastatic forms of these intrinsically aggressive cancers of the head and neck.

## 1. Introduction

The global incidence of head and neck cancer has recently climbed to ~0.74 million new cases per year based on 2015 cancer statistics [1]. With the upper respiratory tract being the first site of contact with environmental carcinogens including certain chemicals (in cigarette smoke or alcohol), air pollutants [2], as well as oncogenic viruses [3,4], the incidence of head and neck cancer will probably continue to rise in the next decade. The collective term of head and neck cancer encompass epithelial malignancies of the oral cavity, tongue, floor of the mouth, pharynx, larynx, oropharynx, nasopharynx, paranasal sinuses, as well as the salivary glands [5,6]. Among which, cancers of the nasopharynx and oropharynx represent two major subtypes with unique and strong etiological association with two distinct oncogenic viruses, namely the Epstein-Barr virus (EBV) and the human papillomavirus (HPV), respectively. The Epstein-Barr Virus (EBV)-associated nasopharyngeal carcinoma (hereafter, abbreviated as EBV(+) NPC) is the most metastatic cancer of the head and neck with the highest prevalence in Southern China and Southeast Asia, while the HPV-associated head and neck squamous cell carcinoma (cancer of squamous epithelial cell origin, hereafter, abbreviated as HPV(+) HNSCC; mostly represented by HPV(+) oropharyngeal cancer) is a rising epidemic cancer of Western countries, including Europe and the US.

For a long time, our understanding on how these oncogenic viruses cause head and neck cancers has remained incomplete. Why are nasopharyngeal and oropharyngeal cancers, which are so close in their anatomical locations, affected by two different DNA viruses? Are there any commonalities in these viral-mediated processes that trigger transformation of the head and neck epithelium? How similar are these two cancers in terms of their underlying biology, which may translate into more effective treatments in the future? Yet, recent genomic characterizations of EBV(+) NPC and HPV(+) HNSCC have revealed multiple new dimensions and insights for the above questions. The big leap in the understanding of the genomic landscapes of these two virus-associated cancers may provide new therapeutic opportunities in the near future.

## 2. Background, Similarities and Differences between EBV(+) NPC and HPV(+) HNSCC

### 2.1. EBV(+) NPC

Nasopharyngeal carcinoma (NPC) is a squamous epithelial cancer arising from the mucosa of the nasopharynx. The nasopharynx is a structure that extends from the skull base to the soft palate’s upper surface, and it connects to the oropharynx [7]. In 2012, a total of 86,691 new cases of NPC were reported worldwide [8], with the highest incidence in endemic regions such as South China and Southeast Asia (especially among the Cantonese population with male ASR (age-standardization rate) of ~13–25/100,000 persons) and moderate rate in North Africa (male ASR of ~4–6/100,000 persons) [9]. In contrast, this unique type of head and neck cancer is rarely found in Western countries, with an incidence rate ~100-fold lower than that in the endemic regions. Interestingly, almost all NPC cases from the endemic regions (>95%) are EBV(+) non-keratinizing undifferentiated carcinoma, while those in non-endemic regions are EBV(−) keratinizing carcinomas with well-differentiated histologic features. The major cause of EBV(+) NPC is believed to be caused by latent infection of the oncogenic virus, EBV. Yet, as of today, it still remains perplexing as to why EBV causes EBV(+) NPC in the endemic regions, but mononucleosis in Caucasians. Unique dietary habits (salted fish consumption), as well as genetic susceptibility factors are likely etiological factors for NPC carcinogenesis. Studies have shown that descendants of emigrants from EBV(+) NPC endemic regions have a higher rate of EBV(+) NPC development even after their settlements in non-endemic regions [10,11,12,13]. This indicates a likely genetic factor behind EBV(+) NPC oncogenesis. The potential strong influence of environmental factors on NPC carcinogenesis was recently revealed by a French study, in which male NPC patients of French origin, but born or living in Maghreb (North Africa) appear to have a 5.7-times higher risk to develop NPC than those of French origin born in France [12]. This study highlights the important impact of environment factors on NPC development. 

EBV(+) NPC is one of the most metastatic head and neck cancers which is often found to metastasize to lymph nodes as well as distant organs at the time of diagnosis. It often metastasizes to the liver, lung and brain for reasons yet to be identified. Pathologically, this intrinsically aggressive cancer is characterized by: (1) high intra-tumoral lymphocyte infiltrations; (2) the presence of EBV genome and expression of EBV latent genes including *EBERs*, *EBNA1*, *LMP1*, *LMP2* and *BARTs*; (3) high levels of circulating EBV DNA in the plasma of NPC patients [14,15]. In fact, recurrences of EBV(+) NPC can be predicted by the increasing levels of plasma EBV DNA in patients [16]. 

Although at early stages (I or II), the curative rate of NPC can be ~90% with the most aggressive concurrent chemoradiotherapy. However, the majority of newly diagnosed cases are of advanced stages (III or IV), and these patients have a dismal 5-year survival of ~50–60%. The high mortality rate of NPC is due to the asymptomatic but aggressive nature of this cancer. Thus far, due to the lack of solid gene-drug knowledge and evidence, no NPC-specific targeted therapy has been approved for NPC treatment. Precision medicine for this cancer has not been developed [17,18,19]. 

### 2.2. HPV(+) HNSCC

HPV(+) HNSCC is largely represented by squamous cell carcinoma (SCC) of the oropharynx, and some other less frequently infected sites, such as the oral cavity, larynx and hypopharynx, etc. [20,21]. Oropharyngeal cancers are caused mainly by persistent infection of the oropharyngeal epithelium by the oncogenic HPV 16 via oral sex [22]. Note that cervical cancer is also caused by HPV 16, as well as HPV 18. The blooming epidemic of HPV(+) HNSCC first noted in the Western countries is starting to be seen in some Eastern countries as well [23]. As of today, as much as 80–100%, and 70% of oropharyngeal cancer from European countries and the US are HPV-positive, respectively [24]. In South Korea and Japan, HPV-associated oropharyngeal cancer is as high as 67% [25], while it remains as low as 15% in China and Hong Kong (where EBV(+) NPC is prevalent) [26]. 

Compared to non-HPV-associated head and neck cancer (named as HPV(−) HNSCC hereafter), HPV(+) HNSCC appears to be more aggressive with a higher tendency to metastasize to distant organs [27]. Standardized conventional treatment modalities are being currently applied for both HPV(+) HNSCC and HPV(−) HNSCC, which include surgery, radiation, chemotherapy (or combination), cetuximab (for all HNSCC), and two newly approved PD-L1 inhibitors (pembrolizumab and nivolumab). Interestingly, with the exception of smokers [28], it is recently noted that HPV(+) HNSCC patients, in general, have better clinical outcomes after treatments than HPV(−) HNSCC patients. This general observation leads to multiple clinical trials aiming at de-intensifying treatments for HPV(+) HNSCC. Although the HPV vaccine has been shown to be effective for the prevention of cervical cancer, and its impact on HPV(+) HNSCC is currently being validated with promising preventive results [29]. Notably, multiple genomic characterization studies of HNSCC, including those for HPV(+) HNSCC, have contributed to new molecular understanding of HNSCC, and opened up new opportunities for precision medicine for HPV(+) HNSCC, as well as HPV(−) HNSCC.

### 2.3. Similarities and Differences between these Two Virally Associated HNCs in General

Theoretically, the exposures to chemical or viral carcinogens are rather similar in these cancers as the nasopharynx and the oropharynx are anatomically connected [30]. Yet, EBV infects the nasopharynx, while HPV infects the oropharynx, giving rise to two distinct cancer types. 

Not often noted, the two cancer types do share some degree of similarities and differences (Table 1): (1) both cancers are etiologically linked with double-stranded DNA viruses (i.e., with DNA genomes), with both EBV and HPV being Group I carcinogens to human (World Health Organization; WHO). Yet, the HPV genome (~8 kb; belonging to the human papillomavirus family) is much smaller than that of the EBV genome (~172 kb; belonging to the Herpesvirus family). The EBV genome found in EBV(+) NPC is episomal and clonal in nature [31], while the HPV genome is mostly found to be integrated into human chromosomes, but also observed to be episomal in some HPV(+) HNSCC cases [32]. (2) Clinically, both cancers are known to have young age onsets at ~40–45 years of age (as compared to 65 years old for HPV(−) HNSCC) [4], and both have a relatively higher male to female predominance of 3 or 4:1. (3) Interestingly, both tumors are small in size and potentially antigenic due to the presence of viral antigens. (4) Both cancers are responsive to some traditional treatments as EBV(+) NPC is sensitive to radiation and chemotherapy, while HPV(+) HNSCCs are known to be treatment sensitive in general. In fact, HPV-positivity has been recognized as a single independent favorable prognostic factor for HNSCC survival [33], even for patients with recurrences or metastases [34]. The treatment sensitivity natures of both cancers are unknown, but it has been postulated that the antigenic viral natures, and the p53 wildtype natures of both cancers may constitute to their treatment responsiveness in general [35]. (5) In terms of cancer diagnosis, both EBV and HPV nucleic acids can be detected in the patient’s body fluids, in addition to the patients’ tumors. EBV DNA can be readily detected in nasal discharge, saliva and plasma of the EBV(+) NPC patients [36,37,38], while HPV DNA can be detected in the saliva and plasma of HPV(+) HNSCC patients (though with limited sensitivity at the moment) [39,40]. (6) Theoretically, both cancers can be potentially prevented by vaccination. Currently, HPV vaccine is already available for HNSCC prevention (as well as cervical cancer prevention), while EBV vaccination has not been successfully developed yet. 

## 3. Genomic Characterizations of EBV(+) NPC and HPV(+) HNSCC Reveal Major Genetic Differences and Similarities

### 3.1. WES and WGS Studies on EBV(+) NPC and HPV(+) HNSCC

As of today, several whole-exome sequencing (WES) and whole-genome sequencing (WGS) studies have been conducted to specifically investigate the genetic aberrations of EBV(+) NPC tumors and HNSCC tumors, which include a small number of HPV(+) HNSCC tumors (Table 2). It has to be noted that the TCGA HNSCC cohort covers only a limited number of anatomic sites in the head and neck region, with only 7 major sites having more than 5% of cases in the cohort. These are cancers of the tongue, larynx, oral cavity, pharynx, lips, floor of the mouth, and the tonsils, etc. As expected from the ethnic and geographical prevalence of these cancers, sequencing and molecular information generated for EBV(+) NPC are largely Asian-relevant, while that for HPV(+) HNSCC are mainly Caucasian-relevant. These findings will be reviewed in details below. 

### 3.2. EBV(+) NPC Has a Relatively Lower Mutational Burden than HPV(+) HNSCC

Three NPC WES studies to date showed that EBV(+) NPC has a relatively lower rate of somatic mutations than HPV(+) HNSCC as well as EBV(+) stomach adenocarcinoma [41,42,43]. This may imply that EBV is performing a reasonable big task on oncogenesis already. In fact, in contrast to the limited viral gene products expressed in HPV(+) HNSCC, EBV does express a number of viral proteins and noncoding RNAs to promote transformation via multiple cellular mechanisms and signaling pathways. With the majority of NPC bearing *TP53* wildtype genes (~94% primary NPC tumors), and the low rate of somatic mutation detected in primary NPC tumors, it appears that early development of NPC does not require the accumulation of many somatic genetic events. During early oncogenesis, transformation of normal nasopharyngeal epithelium (e.g., by activation of telomerase) is believed to be a key process [14]. This is followed by losses of chromosomes 3p and 9p (including inactivation of *RASSF1A* and *CDKN2A* genes that were identified in precancerous lesions of NPC over a decade ago). Such a consistent loss of chr. 3p was recently confirmed by both WGS and WES in 100% cases of >111 microdissected EBV(+) NPC tumors of various stages (Stage I to IV) from Hong Kong, an endemic region in Southeast Asia [43]. It is plausible that both chr. 3p and 9p losses predispose the nasopharyngeal epithelial cells to perisistent EBV latent infection and allows clonal expansion of EBV-infected cells. 

Interestingly, the prognostic value of mutational burden of EBV(+) NPC tumors has been recently examined. We showed that patients with EBV(+) NPC tumors harboring less somatic mutations were found to be associated with better overall survival and disease-free survival than those with higher mutational rates in their tumors [43]. Similarly, patients with HPV(+) HNSCC tumors (also known to have relatively lower mutational rates than HPV(−) HNSCC in general [43,50,52]) are also known to be associated with better clinical outcomes [23]. Though the biological mechanism(s) behind the observed favorable clinical outcomes of these two virally associated head and neck cancers remains largely unknown, plausible mechanisms may include the presence of wildtype *TP53*, and likely immunogenic viral antigens in these cancers.

The reported lower rates of somatic mutation in HPV(+) HNSCC vs. HPV(−) HNSCC are consistent with the fact that HPV has already contributed significantly to HNSCC oncogenesis, and only very few additional somatic mutations are likely needed for tumorigenesis to be full-bloomed. Similarly, a lower rate of somatic mutation was found in HPV(+) vulvar SCC vs. HPV(−) vulvar SCC [53]. However, in a recent Indian WES study on HNSCC patients with heavy carcinogen exposures (i.e., betel-nut or tobacco-chewing), the mutational burden of HPV(+) HNSCC (mainly OSCC) does not differ from that of HPV(−) HNSCCs [48]. This may imply an added mutagenic effect of betel-nut and tobacco-chewing on top of HPV-induced carcinogenesis among Indian HPV(+) HNSCC. 

Thus far, the role of EBV in NPC oncogenesis is still not fully understood, while HPV is believed to contribute to HNSCC oncogenesis via alteration of the activities of two key tumor suppressors, p53 and RB proteins, by HPV E6 and E7 proteins, respectively [54]. In addition to p53 and RB alterations, recent WGS results of 35 HPV(+) HNSCC revealed for the first time that HPV genomic integration into certain important cellular genes, thus affecting their functions, may serve as additional mechanisms for HPV-medicated HNSCC oncogenesis. These include HPV integrations within the *RAD51B* and *ETS2* genes causing their loss-of-function activities, or HPV integration resulting in altered expression of an alternative *CD274* (*PDL1*) transcript [49]. Since viral integration can only be thoroughly studied by WGS, it is believed that larger cohorts of WGS-characterized HPV(+) HNSCC may reveal even more previously unknown oncogenic mechanisms by HPV integration events in HNSCC. As for EBV(+) NPC, it remains to be carefully examined in larger WGS cohorts if there is any EBV integration into the human genome or not. 

### 3.3. EBV(+) NPC and HPV(+) HNSCC Have Different Mutational Signatures

Mutational signature analysis can inform us on the underlying mechanisms of DNA mutations in human tumors. In EBV(+) NPC, mutational analysis showed that spontaneous deamination of 5-methylcytosine (characterized by Signature 2, COSMIC database; implicating an endogenous mutational process) and defective DNA mismatch repair (characterized by co-occurring Signatures 6, 15, 20, and 26) are the possible underlying mechanisms for DNA mutagenesis in this tumor type [41,43]). The presence of Signature 6 in NPC (a signature implying defective DNA mismatch repair and microsatellite unstable tumors) does imply some potential involvement of microsatellite unstable genomic events in NPC mutagenesis. However, the APOBEC-related signature (Signatures 2 and 13) is only found in a small subset of NPC tumors (<20%), implying APOBEC-mediated mutagenesis is not the major event for NPC as compared to defective DNA mismatch repair. Whereas in HPV(+) HNSCC, it is predominated by the APOBEC signature, indicating a mutagenic mechanism due to viral infection, tissue inflammation or retrotransposon activity [55,56]. Chen et al. also reported the APOBEC signature as the major mutational signature for HPV(+) cervical cancer [57]. These findings implicate a clear etiologic contribution by HPV via APOBEC-mediated mutagenesis, which is different from the defective DNA mismatch repair signature contributed by EBV in NPC. These mutational signatures of EBV(+) NPC and HPV(+) HNSCC appear to reflect major differences in viral or environmental carcinogen-driven mutational forces for these cancers.

### 3.4. Overall Comparison of Genomic Profiles between EBV(+) NPC and HPV(+) HNSCC

Shortly after the completion of WES studies on these two virally associated head and neck cancers, it was immediately realized and genomically confirmed that previous scattered reports on low rates of *TP53* and *RB1* mutations are indeed key genomic features of these cancers [41,43,50,52]. More interestingly, independent conclusions from several studies suggest that distinct genomic drives are likely responsible for their tumorigenesis in general. Based on the WES data to date, major “NF-κB activating” somatic aberrations are the key driver events for EBV(+) NPC tumorigenesis, while HPV(+) HNSCC is driven by “PI3K pathway activating” genomic changes [43,50,52,58]. Furthermore, several other important signaling pathways are also genomically altered in subsets of patients with these two cancers.

#### 3.4.1. An NF-kB Genomic Drive for EBV(+) NPC

Prior to the genomic characterization of EBV(+) NPC, various signaling pathways including the EGFR, STAT3, c-MET, NF-κB pathways were believed to be important for NPC tumorigenesis and progression. These previous views were supported by data generated from primary tumors and limited cell line models of EBV(+) NPC. Yet, our recent large scale whole-exome characterization of micro-dissected Asian EBV(+) NPC reveals that NF-κB signaling aberrations are the most critical genomic force driving NF-κB activation in EBV(+) NPC. Over 40% of microdissected EBV(+) NPC tumors were found to harbor genetic aberrations (including translocation, tandem duplication, homozygous deletion and mutation) of negative regulators of NF-κB pathways, which include: *CYLD* (18.6%), *TRAF3* (17.5%), *NFKBIA* (6.7%) and *NLRC5* (4.8%) [43]. *CYLD* is a tumor suppressor gene, when germline mutated, it can contribute to a rare form of inherited syndrome called the CYLD (cylindromatosis) cutaneous syndrome with clinical manifestation of multiple benign tumors in the head and neck region at early age [59]. CYLD is a deubiquitinase that drives the degradation of multiple regulators of the NF-κB pathway [43]. In our study, as high as 18.6% of EBV(+) NPC patient tumors were found to have either inactivating mutations, or gene rearrangements of *CYLD* with high allele frequencies. Somatic mutation of *CYLD* was also reported in another exome and target-sequencing study [42]. CYLD restoration in a CYLD-deficient NPC cell line, C666-1, confirmed its tumor-suppressive role in EBV(+) NPC cell context. On the other hand, ectopic expression of *CYLD* mutants was found to elevate NF-κB activity in NPC cells, thus demonstrating a mutational drive for NF-κB signaling by *CYLD* aberrations in NPC. In particular, activation of the atypical NF-κB pathway (as demonstrated by a marked increase in p50/p50/Bcl-3 nuclear complex) appears to be the main consequence driven by *CYLD* mutants in the EBV(+) NPC cell context [43]. It is note-worthy that CYLD serves multiple functions in normal and pathophysiology, it is plausible that *CYLD* mutations may affect other functions in EBV(+) NPC cells, such as cilia formation and innate immunity, which is consistent with the loss of ciliated structures during NPC development [60].

In addition to CYLD, another major negative regulator of NF-κB, namely TRAF3 (TNF receptor associated factor 3), was also found to be aberrant in as high as 17.5% of microdissected EBV(+) NPC tumors [43]. We also demonstrated that *TRAF3* WT gene inhibited the non-canonical NF-κB signaling in NPC cells, while *TRAF3* mutants lost the ability to suppress NF-κB activation in EBV(+) cell context. These genomic aberrations may cause constitutive activation of the NF-κB signaling pathway. These genome findings do suggest the importance of NF-κB targeting for EBV(+) NPC. Microdissection, which aims at enriching tumor cell content prior to sequencing, appears to be crucial for accurate identification of NPC somatic changes as heavy stromal content including lymphocyte infiltration can obscure genomic findings. Without microdissection, the detected frequencies for *TRAF3*, *NFKBIA* and *NLRC5* mutations were much lower [41,42]. Nevertheless, similar to EBV(+) NPC, a subset of HNSCC with episomal HPV infection also harbors frequent somatic *CYLD* and *TRAF3* mutations [61].

In addition to *CYLD* and *TRAF3* aberrations, an EBV oncoprotein, the latent-membrane protein 1 (LMP1) is also known to be a potent activator for NF-κB signaling [62]. LMP1 has been shown to phosphorylate the IKK complex and induce IκBα degradation, thus resulting in constitutive NF-κB activation. Our exome study revealed for the first time that LMP1 overexpression and genomic aberrations of NF-κB pathway are mutually exclusive [43]. In sum, the LMP1-overexpressing NPC subset and the NF-κB altered NPC subset together account for >70% of all EBV(+) NPC cases. Thus, NF-κB activation is selected for, by both somatic and viral events during NPC pathogenesis in a large majority of patients. This is consistent with the finding that non-coding RNAs of EBV, called *Epstein-Barr virus* (*EBV*)-encoded small RNAs (*EBERs*) also function to promote NF-κB signaling via their interactions with TLR3 [63]. It has been proposed that LMP1 and EBERs form a regulatory loop feeding forward to activate NF-κB signaling for inflammatory responses in NPC.

In summary, multiple genomic and viral events aim to constitutively activate NF-κB signaling for cell proliferation, survival, and inflammation during NPC tumorigenesis. For the very first time, this unique NPC/NF-κB genomic signature unifies many previous scattered findings for this cancer. Whether such a unique NF-κB genomic landscape drives only the non-canonical NF-κB pathway or not, and whether it can help sustaining EBV infection and tumor growth will require further investigations. Yet, targeting of these NF-κB aberrations for NPC treatment development will require multiple representative EBV(+) NPC models, which are currently lacking.

#### 3.4.2. A Prominent PI3K Genomic Drive for HPV(+) HNSCC

HPV is the causative agent of cervical cancer, genital warts, oral papillomas, HPV(+) HNSCC, and some benign tumors. The HPV family has more than 150 subtypes, and many of them have been classified as high-oncogenic risk subtypes, including HPV 16, 18, 31, 33, 35, 39, 45, 51, 52, 56, 58, and 59 for tumorigenesis [64,65]. In particular, HPV 16 and 18 have been the focus of study in cervical cancer. It has been well-recognized that the transforming activities of HPV 16 and HPV 18 are largely exercised through the E6 and E7 oncoproteins, which impair two most important tumor suppressors in mammalian cells. E6 targets p53 for proteasome degradation, resulting in cell cycle dysregulation and the loss of p53-mediated apoptosis in HPV infected cells. Whereas E7 binds to and inactivates RB1, and promotes RB1 degradation. This E7-mediated RB1 degradation results in the release of activated E2F transcription factor, which promotes S phase gene transcription, including that of the p16INK4a (or *CDKN2A* or p16). Thus, cell cycle progression is dysregulated by E6 and E7 proteins upon HPV infection. Furthermore, HPV integration into the human chromosomes can result in E6 and E7 amplification via viral-host concatemers in HPV(+) HNSCC tumor samples [32].

Prior to genomic characterization, HNSCC collectively, was believed to be governed by several major growth and survival pathways such as EGFR, JAK/STAT signaling, and p53 aberrations. Yet, the recently completed WES studies revealed a wide array of genomic aberrations affecting growth, differentiation, senescence, apoptosis, DNA repair, and immunity in HNSCC. These include genomic aberrations of *TP53*, *CDKN2A*, *HRAS*, *NOTCH*, *PIK3CA*, *PTEN* and *CASP8*, etc. [50]. Yet, with only 36 HPV(+) HNSCC being exome characterized by the TCGA [50] and reported by us [52], HPV(+) and HPV(−) HNSCCs immediately emerged as distinct types of HNSCC based on their differential genomic profiles. The distinct genomic profile of HPV(+) HNSCC has then been at least partially confirmed in several targeted sequencing studies [66,67,68] and is expected to be validated in even larger cohorts as there are many more incidences of HPV(+) HNSCC in Western countries. Yet, based on the published WES data of 36 HPV(+) HNSCC tumors from the TCGA, HPV(+) HNSCC were found to have almost no (or <1%) somatic *TP53* mutation, while HPV(−) HNSCC were characterized by a very high rate of *TP53* mutations (~85%; 206/243 cases). This particular low rate of *TP53* mutation in HPV(+) HNSCC may simply reflect a “sufficient and efficient” p53-degradating activity of E6 protein on the infected epithelium during HPV-mediated HNSCC oncogenesis. Interestingly, Zevallos et al. reported a previously un-noticed effect of heavy smoking on HPV(+) HNSCC genomic profiles [69]. Zevallos et al. found that HPV(+) HNSCC tumors deriving from heavy smokers (>10 pack-years) have exclusive mutational events of *TP53*, *CDKN2A*, *KRAS* and *NOTCH1*, and have worse survival than those HPV-positive patients with <10 year-pack year of smoking history. This may suggest the added effects of smoking, on top of HPV, on HNSCC genomic landscapes, which may have future clinical implications, including biomarker development.

Another unexpected genomic finding is the presence of a relatively high rate of *PIK3CA* mutation and amplification in HPV(+) HNSCC (~40–50% of cases). As first reported by us [52], *PIK3CA* somatic aberrations were actually found to be 2 folds higher in HPV(+) HNSCC than that of the HPV(−) HNSCC. This is similar to the high rate of *PIK3CA* mutations in HPV(+) cervical cancer (39–41% cases; TCGA; www.cbioportal.org), implicating the biological importance of PI3K activation in these HPV-positive cancers. A recent study by Han et al. also demonstrated the presence of *PIK3CA* mutations in HPV(+) vulvar SCC [70].

Most of the identified HNSCC-associated *PIK3CA* mutations are hotspot mutations [71], and drivers for HNSCC cell growth [52]. By far, *PIK3CA* hotspot mutations rank the first among all recurrent mutations in HPV(+) HNSCC. These include: *PIK3CA* R88Q, E542K and E545K. It is possible that these gain-of-function *PIK3CA* activating genomic events in HPV(+) HNSCC may promote PI3K pathway activation, thus cell survival, growth, or perhaps even immune evasion [72].

It was recently speculated that *PIK3CA*-mutated HPV(+) HNSCC may confer sensitivity of these tumors for PI3K inhibitors. Yet, Brand et al. showed that some HPV(+) HNSCC cell models, and patient-derived xenografts (PDXs) could be resistant to the PI3K pathway inhibitor, BYL-719 [73]. Subsequent mechanistic investigation showed that PI3K targeting in HPV(+) HNSCC cells could result in a feedback upregulation of ERBB3 signaling via E6 and E7 induction, thus conferring PI3K resistance. Importantly, it was then demonstrated that co-targeting of PI3K and ERBB3 could be an effective approach circumventing this ERBB3 feedback, thus resulting in good antitumor efficacy for HPV(+) HNSCC [74,75]. 

#### 3.4.3. Systematic Pathway Comparisons between EBV(+) NPC and HPV(+) HNSCC Reveal Commonalities in NF-κB and PI3K Pathway Activation in Both Cancers

The original messages from the WES studies are rather clear that EBV(+) NPC is a NF-κB-driven cancer, while HPV(+) HNSCC is a PI3K-driven cancer. Yet, in this review, based on the pathways that were found to be important for both cancers, we re-analyzed the WES data and systematically compared all key pathways in both cancers to better comprehend how similar or how different they are genomically. (Note that we only include the genomic data of HPV(−) oropharyngeal cancer for reference purposes as HPV(+) HNSCC is mostly represented by cancer arising from the oropharynx). 

**Figure 1:**
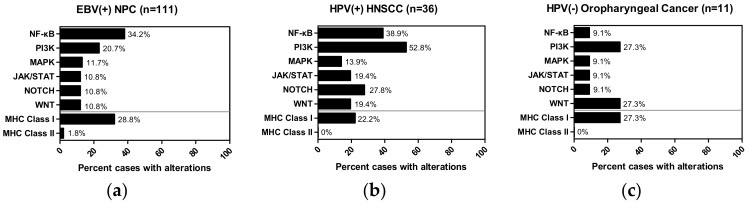
Bar graphs showing percentage of tumors harboring somatic mutations of 6 major signaling pathways (NF-κB, PI3K, JAK/STAT, MAPK, NOTCH, WNT), and of the MHC Class I, and MHC Class II genes in (**a**) EBV(+) NPC cohort (*n* = 111), (**b**) HPV(+) HNSCC cohort (*n* = 36; TCGA) and (**c**) HPV(−) oropharyngeal cancer cohort (*n* = 11; TCGA). The NF-κB pathway is defined as *TAB1/2/3*, *MAP3K7/14*, *CHUK*, *IKBKB*, *IKBKG*, *NFKBIA*, *NFKBIE*, *REL*, *RELA/B*, *NFKB1/2*, *LTBR*, *TNF*, *TNFAIP3*, *TNFSF11/13B*, *TNFRSF1A/8/11A/13C*, *BTRC*, *CYLD*, *NLRC5*, *TRADD*, *CD40*, *CD40LG*, *LTA*, *TRAF2/3/5/6*, *IL1B* and *IL1R1.* The PI3K pathway is defined as *AKT1/2/3*, *PIK3CA/B/D/G/2A/2B/2G*, *PIK3AP1*, *PIK3IP1*, *PDK1*, *MTOR*, *TSC1*, *TSC2*, *PTEN*, *RICTOR*, *RPTOR*, *RHEB* and *PIK3R1/2/3/4/5/6*. The JAK/STAT pathway is defined as *JAK1/2/3*, *STAT1/2/3/4/5A/5B/6*, *PTPN11*, *IL6*, *IL6R*, *IL6ST and SOCS3*. The MAPK pathway is defined as *SHC1/2/3*, *GRB2*, *H/N/KRAS*, *A/BRAF*, *RAF1*, *MAP2K1/2*, *MAPK1/3*, *RPS6KA1* and *DUSP1/2/3/4/5/6/7/9*. The NOTCH pathway is defined as *DLL1/3/4*, *JAG1/2*, *NOTCH1/2/3/4*, *NUMB*, *DTX1/3L*, *NEDD4*, *MAML1*, *RBPJ*, *POFUT1*, *HES1/5* and *HEY1/2/L*. The WNT pathway is defined as *WNT1/3A/5A/5B/7A*, *CTNNB1*, *HNF1A*, *FZD1/2/3/7/8/9/10*, *AXIN1*, *LEF1*, *LOXL2*, *DVL2/3*, *NKD1/2*, *TAB1/2*, *GSK3B*, *CSNK1A1*, *NLK* and *LRP5/6*. The MHC Class I genes are defined *as HLA-A/B/C/E/F/G/H/K/L/J*, *B2M* and *NLRC5*. The MHC Class II genes are defined *as HLA-DMA/B*, *HLA-DOA/B*, *HLA-DPA1/A2/B1/B2*, *HLA-DQA1/A2/B1/B2/B3* and *HLA-DRA/B1/B2/B3/B4/B5/B9*.

As shown in Figure 1, apart from NF-κB aberrations (34.2% cases) that was published to be the most frequently altered pathway, PI3K pathway mutations rank the second most commonly mutated signaling pathway in EBV(+) NPC (20.7% cases), followed by the MAPK pathway (11.7% cases), and JAK/STAT, NOTCH, WNT (all with 10.8% cases mutated). Interestingly, in HPV(+) HNSCC, aberrations of the PI3K pathway (52.8% cases; previously published) was seconded by aberrations of the NF-κB pathway genes (38.9% cases) [50] (Figure 1), followed by aberrations of the NOTCH (27.8% cases), JAK/STAT (19.4% cases), WNT (19.4% cases), and the MAPK (13.9% cases) pathways. As for HPV(−) oropharyngeal cancer, which does appear to be mutationally different from the HPV(+) HNSCC, the most common aberrations are that of the PI3K pathway, WNT pathway, as well as the MHC Class I genes (all are noted in 27.3% cases respectively). Therefore, NF-κB and PI3K pathways are, in fact, the top two most commonly mutated signaling pathways in these two virally associated head and neck cancers (Figure 1a and b). Furthermore, as far as MHC Class I/II genes are concerned, both of these virally associated cancer types, as well as HPV(−) oropharyngeal cancer, all showed relatively higher rates of mutations of MHC Class I gene than that of MHC Class II genes (Figure 1). This suggests that MHC Class I gene defects can be more important and relevant for both viral and non-viral mediated tumorigenesis or disease progression. Yet, particularly for EBV(+) NPC and HPV(+) HNSCC, it remains to be determined if defective viral antigen presentation via MHC Class I-dependent pathway is involved in their carcinogenesis. (Figure 1). 

As far as the PI3K pathway is concerned, mutations of the well-known oncogene, *PIK3CA*, occur far more frequently in HPV(+) HNSCC than in EBV(+) NPC (Table 3; 36% cases vs. 4% cases, respectively). Interestingly, in microdissected NPC, *PTEN* mutation is the most common event of the PI3K aberrations (5/28 PI3K mutated tumors), followed by mutations of a diverse group of PI3K players, such as *PIK3CA* (4/28), *PIK3C2G* (4/28), *MTOR* (2/28), *PIK3R4* (2/28), *PIK3AP1* (2/28)., *PIK3CB/G* (2/28 each), *TSC1* (2/28) and *RICTOR* (2/28). In fact, some of these recurrent mutations have also been identified in another NPC exome study in Asia [*PIK3CA* (1/56), *MTOR* (1/56), *PIK3C2G* (1/56) and *PIK3CG* (1/56)] [41]. Note that *PIK3CA* mutations only occur in a small subset of HPV(−) oropharyngeal cancer (9% cases) as compared to HPV(+) HNSCC (36% cases).

Whilst for the NF-κB pathway, *CYLD* and *TRAF3* mutations appear to occur at a similar high rate in both cancers (8–11% rates), followed by the same rate of mutations of *NLRC5* (6% rates) (Table 3). This may suggest that *CYLD*, *TRAF3* and *NLRC5* aberrations are critical for oncogenesis for a major subset of these cancers. Interestingly, mutations of *NFKBIA*, an inhibitor of the NF-κB /Rel A complex is only observed in EBV(+) NPC (7/111, 6%), but not in HPV(+) HNSCC. Note that *CYLD*, *TRAF3* and *NLRC5* are not found in HPV(–) oropharyngeal cancer (Table 3). In sum, a “favorable NF-κB niche” is perhaps permissive or advantageous for both EBV and HPV-associated cancer cells to grow, survive, and evade the immune surveillance.

Enrichment of *PIK3CA* mutations in HPV(+) HNSCC was first noted in WES studies [50,52], which was later confirmed by another targeted sequencing study of 51 cases of mostly advanced HPV(+) HNSCC (617 cancer-associated genes [76]). This study also revealed enrichments of *MLL3*, *DDX3X*, *FGFR2*, *FGFR3*, *NOTCH1*, *NF1*, *KRAS* and *FBXW7* mutations in this cancer. Further signaling network analysis indicated a connection with aberrations in DNA damage, FGF/FGFR, JAK/STAT and immune genes (*HLA-A*, *HLA-B*) [76]. Interestingly, some of these genes are also found to be altered in EBV(+) NPC, especially those related to antigen presentation. In particular, subsets of EBV(+) NPC tumors do harbor loss-of-function of MHC Class I genes (such as *HLA-A*, *HLA-B*, *HLA-C*, *B2M*, *NLRC5*) [43]. It is worth-noting that NPC patients with somatic MHC Class I gene defects do have poorer overall survival and disease-free survival [43]. Further, germline MHC haplotypes, HLA-A2 and HLA-B46, have also been previously shown to be associated with EBV(+) NPC susceptibility in familial and population studies of NPC [77]. These cumulative findings indicate a key role of MHC genes in immune evasion for EBV(+) NPC. Similarly, such an immune evasion mechanism, predominantly offered by MHC class I gene aberrations, is likely to be active in HPV(+) HNSCC as well.z

Although the intricate interactions between the viral genomes and somatic events initiating/ facilitating oncogenesis of these two head and neck cancers are incompletely understood, the central message is becoming much clearer than before. In both cancers, two major signaling pathways are likely critical for, or in support of an inflammatory (NF-κB) and survival-promoting (PI3K) environment for full-bloom oncogenesis.

Another similarity between these two virally associated cancers is that they seem to employ both specific somatic (genetic) and viral events for the achievement of a common goal, i.e. to constitutively activate a critical signaling pathway that is essential for their oncogenesis. This is apparent in EBV(+) NPC, as both high rates of NF-κB pathway mutations and LPM1 overexpression (which also drives NF-κB activation) are found to be existing in mutually exclusive subsets of NPC tumors, totally more than 70% of all NPC cases. These findings do strongly imply that both somatic and viral events are exerting common efforts driving constitutive NF-κB activation during NPC carcinogenesis [43]. Similarly, a very high rate of PI3K pathway mutations (>50–60% cases) in HPV(+) HNSCC clearly implicates the importance of PI3K activation in HPV-mediated HNSCC oncogenesis. In fact, this is supported by previous findings that HPV-16 E7 oncoprotein is a driver for PI3K pathway activation [78], highlighting the biological importance of PI3K activation during HPV-mediated carcinogenesis of the head and neck. Yet, whether PI3K mutations and the expression levels of E7 oncoprotein in HPV(+) HNSCC patient tumors also follow a mutually exclusive relationship (as NF-κB aberrations and LMP1 overexpression in NPC) remains to be examined. 

The questions remaining are: Would these pathway activations serve to facilitate viral-mediated oncogenesis that are critical for both EBV(+) NPC and HPV(+) HNSCC? How do the NF-κB defects (e.g. *CYLD*, *TRAF3* aberrations) and the immune system defects (including MHC loss) lead to tumor formation, or immune evasion (e.g., via dysregulation of innate and adaptive immune responses) [79,80]?

## 4. Limited Genomic Profiles of EBV(+) NPC and HPV(+) HNSCC Recurrences and Metastases

With the understanding of somatic alterations in a reasonable number of primary NPC tumors, there are limited genomic information for recurrent or metastatic NPC. Thus far, there are reported somatic changes of 11 local recurrences and 22 metastatic EBV(+) NPC tumors only [43]. Interestingly, similar predominant mutation signatures were found in both primary and recurrent tumors, suggesting the absence of any new or drastically different etiological factors during NPC recurrences or metastasis. As mutation rate is concerned, *TP53* mutations were identified to be >2-fold enriched in recurrent or metastatic tumors (15.2% cases with *TP53* mutation) vs. primary tumors (6.4% mutation rate of *TP53*). This appears to support the notion that NPC clones with genomic instability are selected for during progression to more advanced diseases.

In addition to *TP53* mutations, NPC patients bearing PI3K/MAPK pathway-mutated recurrent and metastatic tumors were found to have significantly poorer overall survival than those with WT tumors [43]. These PI3K/MAPK pathway mutations include *NRAS*, *KRAS*, *PIK3CA*, *PTEN*, *NF1*, *TSC1* and *FGFR3*. In two of the three pairs of primary NPC and local recurrence samples, *RAS* activating mutations were shown to be progression drivers. Activations of the MAPK and PI3K-AKT-mTOR signaling are known to control multiple cellular processes and promote resistance to anticancer therapies in human cancers. Therefore, it is plausible that tumors with acquired activating *RAS* mutations can cause recurrences via resistant mechanisms to chemotherapy and radiotherapy through MAPK pathway activation. The WES data thus far revealed a noticeable frequency of 5% tumors with inactivating *PTEN* mutations, and 4% cases with *PIK3CA* hotspot mutations and 20% cases with *PIK3CA* amplification. Whether these PI3K aberrations can contribute to therapy resistance and subsequent disease recurrences remain unknown. Lastly, recurrent mutations of *ABL1*, *BUB1B*, *NCOR1*, *CARS*, *HSP90AB1*, and *NCOA1* were uniquely found in recurrent or metastatic NPC tumors, but not in primary NPC. Yet, it remains to be further validated if these unique mutations are determinant of NPC progression or not. Future genomic investigations on larger recurrent and metastatic NPC cohorts will allow more accurate delineation of critical drivers for NPC progression and recurrences for both prognosis and treatment advancement. 

When compared to NPC, the genomic landscape of recurrent or metastatic HPV(+) HNSCC is even more obscure. The most comprehensive TCGA cohorts [50] as well as the recently completed larger cohort only represent the landscape of primary tumors, but not that of recurrences or metastasis. Currently, there is no reported comprehensive exome data on recurrent and metastatic HPV(+) HNSCC. This is largely due to the rarity of recurrent HPV(+) HNSCC, as HPV-positivity is associated with favorable outcomes. In short, the recurrent and metastatic genome of these two virally associated head and neck cancers are largely unknown. Yet, it is believed that the ultimate answers for curative treatments do lie partially on these important genomic information, as recurrent and metastatic forms of these tumors are mostly fatal.

## 5. Implications for Precision Medicine Development

Based on the survival data of a medium size cohort of 111 EBV(+) NPC cases with primary tumors, several major druggable events and genetic biomarkers have emerged [43]. In contrast, the small HPV(+) HNSCC cohort of 36 cases appears to be limited for biomarkers discovery, though some druggable events can be identified. 

The genomic data for both cancer types reveal a promising druggable target, the fibroblast growth factor receptor 3 (*FGFR3*). *FGFR3* is found to be mutated or rearranged in 11–14% of HPV(+) HNSCC (4/36 cases) [50,76] vs. 0% of HPV(−) HNSCC (0/243 cases; TCGA), and in 1.8% of EBV(+) NPC (2/111 cases) [81]. In particular, the *FGFR3-TACC3* gene rearrangement has been identified in many other tumor types, including cervical cancer (1.3–1.9%; [82]), lung cancer (1.9%; [83]), esophageal cancer (2.1%) [84], lung adenocarcinoma [85], bladder cancer [86], endometrial adenocarcinoma, gall bladder carcinoma, non-small cell lung cancer (NSCLC), pancreatic exocrine carcinoma, renal cell carcinoma [87], etc. Importantly, the druggability of *FGFR3* aberrations has been demonstrated in *FGFR3-TACC3* rearranged glioma, bladder and cervical cancer cells, which were found to have high sensitivity to FGFR3 inhibitors [88,89,90]. Interesting, the sensitivity of *FGFR3-TACC3* rearranged cervical cancer cells towards FGFR3 inhibition (BGJ398) was found to be dependent on the wildtype PI3K status of the cells [82].

In cervical epithelial cell models, expression of the *FGFR3-TACC3* fusion results in transformation to squamous cell carcinoma. Similarly, such a fusion gene has oncogenic or transforming activities in glioblastoma [91], and nasopharyngeal cancer [81], as well as in the Ba/F3 cell model [85]. Recent findings in head and neck cancer show that *FGFR3-TACC3* fusion is capable of substituting for EGFR signaling in HNSCC models, and confers resistance to EGFR targeting in HNSCC and possibly in other cancers [92]. In fact, subsequent studies showed that such a fusion gene can confer resistance to all three generations of EGFR tyrosine kinase inhibitors in *EGFR*-mutated NSCLC [93]. It is believed that this fusion protein can substitute EGFR signaling and may act to promote inflammation in the cancer site as well. The clinical efficacy of targeting such as a fusion is currently under investigation.

In addition to FGFR3, other prominent drug targets include: BRCA1/2, SRC, LYN, YES1, ALK, VEGFA, EGFR, FGFR1/2/3/4, ERBB2/3/4, PDGFRA/B, JAK1/2/3, NTRK1/2/3, MET, PIK3CA, TSC1/2, MTOR, H/NRAS, BRAF, MAPK1/3, MAP2K1/2, NF1, NR2C2, PTPRD/T, PDCD1 and CD274. (EBV(+)NPC 36/111, 32%; HPV(+)HNSCC 21/36, 58%). Although these potentially druggable targets are mutated in EBV(+) NPC and HPV(+) HNSCC at notable but different frequencies, their druggabilities in patients remain to be investigated. Preclinical PDXs results are supportive of *PIK3CA*-mutated HNSCC for PI3K pathway inhibitors [52]. Clinical trials are ongoing to determine the clinical efficacy of PI3K targeting in HNSCC in general, including HPV(+) HNSCC. It still remains to be examined if mutations of other major players of the PI3K pathway (e.g., MTOR, TSC1/2) can also confer sensitivity to PI3K/mTOR inhibitors in HPV(+) HNSCC or not. Overall, there is a sizeable subset of HNSCC patients with receptor-tyrosine kinase (RTK) mutations (e.g., *NTRK1/2/3*, *ERBB1/2/3/4*, *PDGFRs*, *MET*, etc.) and it remains to be carefully defined if these RTK mutations will cause any drug sensitivity in preclinical models or in patients. Yet, *ERBB1* (*EGFR*) mutations are not likely to contribute to responses in most human cancers besides demonstrated efficacies in NSCLC. 

It is important to realize that an integrative genomic-proteomic approach for drug target discovery is gaining practical importance in precision medicine development. Several such studies discovered new links between *PTPRT* and *PTPRD* mutations and STAT3 activation in HNSCC patient tumors and preclinical models. These findings strongly imply a potential druggability of *PTPRD-* and *PTPRT*-mutant tumors with STAT3 inhibitors [94,95]. Lastly, results are anticipated to see if *HRAS* mutations can confer sensitivity to the new RAS inhibitor, tipifarinib in clinical trial settings or not. 

## 6. Implications for Immunotherapy

**Figure 2:**
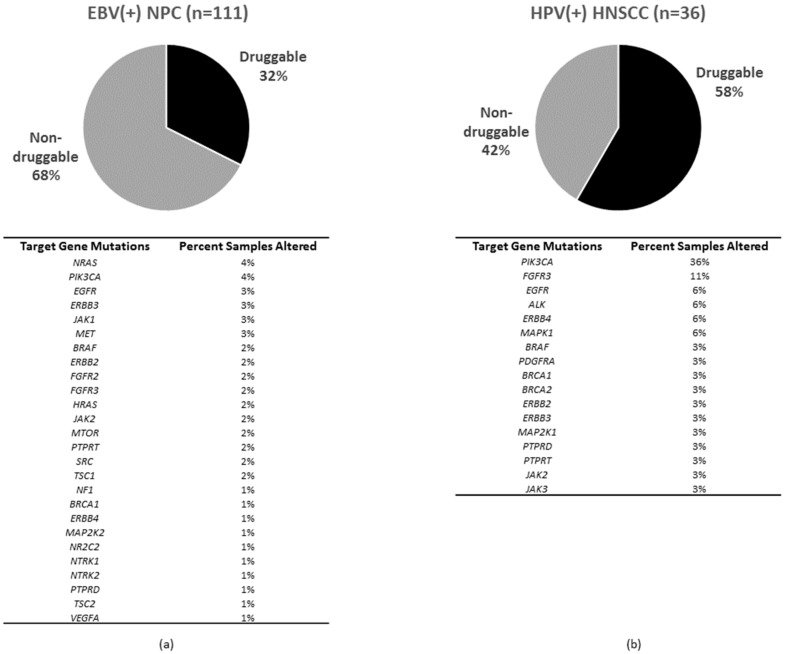
Figure showing percentage of tumors harboring at least one targetable event in (**a**) EBV(+) NPC cohort (*n* = 111) and (**b**) HPV(+) HNSCC cohorts (*n* = 36; TCGA). The targetable genes include: *BRCA1/2*, *SRC*, *LYN*, *YES1*, *ALK*, *VEGFA*, *EGFR*, *FGFR1/2/3/4*, *ERBB2/3/4*, *PDGFRA/B*, *JAK1/2/3*, *NTRK1/2/3*, *MET*, *PIK3CA*, *TSC1/2*, *MTOR*, *H/NRAS*, *BRAF*, *MAPK1/3*, *MAP2K1/2*, *NF1*, *NR2C2*, *PTPRD/T*, *PDCD1* and *CD274 (PDCD1LG1).* The percent cases with targetable genes mutation in each cohort are shown below each pie chart.

The genomic profiles of both EBV(+) NPC and HPV(+) HNSCC alert us that there are subsets of patients bearing tumors with antigen-presentation defects. In EBV(+) NPC, as high as 28.8% of patients have MHC Class I gene aberrations (Figure 1), and this subgroup of patients was found to have a poorer overall survival with conventional chemotherapies and/or radiation therapies, when compared with the MHC Class I wildtype patients [43]. This finding suggests that an immune component, such as defective antigen presentation, may be one underlying reason for poor survival in NPC. In fact, a recent study by Ma et al. showed that an EBV(+) NPC patient with aggressive recurrence had a complete response to a PD-L1 inhibitor, Nivolumab [96], highlighting the potential impact of an active immune recognition in NPC treatment response and outcome. Similar to findings in most other cancers, the predictive biomarkers behind such a dramatic response to checkpoint inhibitors in EBV(+) NPC is still unknown. Whether MHC defects in NPC affect the efficacies of immunotherapies remain to be examined carefully. Similarly, as high as 22.2% cases of HPV(+) HNSCC harbor MHC Class I gene aberrations (Figure 2). It is likely that HPV(+) HNSCC tumors may adopt this potential MHC defect for immune evasion. Again, the potential impact of these MHC losses on PD-L1 targeting in HPV(+) HNSCC are unknown. As mentioned earlier, both EBV(+) NPC and HPV(+) HNSCC can express viral antigens in addition to tumor neoantigens arising from somatic mutations. Whether these “extra” viral antigens may act like neo-antigens (as in MSI-high tumors), and be able to confer better clinical outcome in patients towards checkpoint inhibitors may worth future investigations.

The role of *PDL1* gene rearrangement on head and neck cancer response to PD-L1 targeting remains undefined. Recently, Bellone et al. reported an exceptional pembrolizumab responder with *PDL1-rearranged* metastatic ovarian cancer [95]. Although a *PD-L1*-rearranged HPV(+) HNSCC case has recently been reported [97], the ability of such an aberration in conferring pembrolizumab sensitivity to HNSCC remains unclear. A better understanding of responder genetics for immune checkpoint inhibitors in both clinical and preclinical models will facilitate the use of immune checkpoint inhibitors for all head and neck cancers, including both EBV(+) NPC and HPV(+) HNSCC. 

## 7. Conclusions

In conclusion, the completion of WES characterization of a reasonable number of EBV(+) NPC and a small number of HPV(+) HNSCC tumors has already contributed to a big leap to the understanding of these virally associated head and neck cancers. Our next step is taking these genomic data on to identify key driver events for treatment exploration. Functional annotations of these potential drivers and druggable genetic events will greatly facilitate rapid translation of these genomic findings into clinics. Lastly, the cure of these often aggressive cancers may actually lie in future collaborative endeavors to comprehensively characterize the genomics of large and representative cohorts of recurrent and metastatic EBV(+) NPC and HPV(+) HNSCC.

## Figures and Tables

**Table 1:** Similarities and differences between EBV(+) NPC and HPV(+) HNSCC.

Features	EBV(+) NPC	HPV(+) HNSCC
Site	Nasopharynx	Mainly orapharynx
Onset age	Early young (~45)	Early young (~40–45)
Oncovirus infection	Herpesvirus, EBV (~172 kb), clonal, episomal	Human papillomavirus, HPV16(~7–8 kb), 50–70% intergraded, 30–50% episomal
Tumor size	Small	Small
Major tumor cell type	Most non-keratinizing squamous cell carcinoma	Squamous cell carcinoma (SCC)
Clinical outcome	Poor at advanced stages	Favorable
Treatment sensitivity	Sensitive	Sensitive

EBV(+) NPC, Epstein-Barr virus-positive nasopharyngeal carcinoma; HPV(+) HNSCC, Human papillomavirus-positive head and neck squamous cell carcinoma.

**Table 2:** WES or WGS studies for NPC and HNSCC.

	Publications	Ethnicity	WES or WGS
**NPC**	Lin, et al. [41]	Asian (*n* = 61)	56 tumors and 5 cell lines (WES)
	Zheng H, et al. [42]	Asian (*n* = 59)	51 tumors and 8 recurrent tumors (WES)
	Li, et al. [43]	Asian (*n* = 97) Caucasian (*n* = 8)	111 micro-dissected EBV(+) NPCs from 105 unique subjects (WES, and 15 NPCs with WGS)
	Li Zhang, et al. [44]	Asian (*n* = 111)	111 EBV(+) NPCs (WES)
	Yock Ping Chow, et al. [45]	Asian (*n* = 10)	10 tumors
**HNSCC**	Nicolas Stransky, et al. [46]	Caucasian (*n* = 92)	92 tumors (7X WES with 14 HPV(+) HNSCC, 2 tumors with WGS)
	Nishant Agrawal, et al. [47]	Caucasian (*n* = 32)	32 tumors (WES; 4 HPV(+) HNSCC)
	India Project Team of the International Cancer Genome Consortium [48]	Asian (*n* = 50)	50 tumors (WES; 13 HPV(+) HNSCC)
	Micheal Parfenov, et al. [49]	Caucasian (*n* = 150)	150 tumors (WES; WGS, 35 HPV(+) HNSCC)
	TCGA [50]	Caucasian (*n* = 279)	279 tumors (WES; 36 HPV(+) HNSCC)
	Matthew L. Hedberg, et al. [51]	Caucasian (*n* = 23)	23 tumors (WES; 1 HPV(+) HNSCC)
	TCGA (provisional)	Caucasian (*n* = 530)	530 tumors (WES)

Whole-exome sequencing (WES) or whole-genome sequencing (WGS).

**Table 3:** Tables showing the percentage of tumor with somatic mutations in the most affected NF-κB pathway and PI3K pathway in EBV(+) NPC, HPV(+) HNSCC and HPV(−) oropharyngeal cancer, respectively.

NF-κB Pathway Aberrations				PI3K Pathway Aberrations			
Gene	EBV(+) NPC (*n* = 111)	HPV(+) HNSCC (*n* = 36)	HPV(−) Oropharyngeal Cancer (*n* = 11)	Gene	EBV(+) NPC (*n* = 111)	HPV(+) HNSCC (*n* = 36)	HPV(−) Oropharyngeal Cancer (*n* = 11)
*CYLD*	10%	11%	0%	*PTEN*	5%	3%	0%
*TRAF3*	8%	11%	0%	*PIK3C2G*	4%	3%	0%
*NLRC5*	6%	6%	0%	*PIK3CA*	4%	36%	9%
*NFKBIA*	6%	0%	0%	*MTOR*	2%	0%	0%
*CHUK*	2%	0%	0%	*PIK3AP1*	2%	0%	9%
*DTL*	2%	0%	0%	*PIK3CB*	2%	3%	0%
*IL1B*	2%	0%	0%	*PIK3CG*	2%	3%	0%
*BTRC*	1%	0%	0%	*PIK3R4*	2%	0%	0%
*CD40LG*	1%	0%	0%	*RICTOR*	2%	3%	0%
*IKBKG*	1%	0%	0%	*TSC1*	2%	0%	0%
*IL1R1*	1%	3%	0%	*AKT2*	1%	0%	0%
*LTBR*	1%	3%	0%	*PIK3C2A*	1%	0%	0%
*NFKB2*	1%	0%	0%	*PIK3CD*	1%	3%	0%
*REL*	1%	0%	0%	*PIK3R1*	1%	3%	0%
*RELA*	1%	0%	0%	*PIK3R5*	1%	3%	0%
*TAB1*	1%	6%	0%	*RPTOR*	1%	3%	9%
*TAB3*	1%	3%	0%	*TSC2*	1%	0%	0%
*TNF*	1%	0%	0%	*AKT1*	0%	3%	0%
*TNFAIP3*	1%	3%	0%	*PDK1*	0%	3%	0%
*TNFRSF1A*	1%	0%	0%	*PIK3R3*	0%	3%	9%
*TNFRSF13C*	0%	3%	0%	*PIK3R6*	0%	3%	0%
*TRAF5*	0%	3%	0%				
*MAP3K7*	0%	3%	0%				
*TNFRSF13B*	0%	0%	9%				

## Abbreviations

The following abbreviations are used in this manuscript:

EBV	Epstein-Barr virus
EBV(+)	Epstein-Barr virus-positive
NPC	Nasopharyngeal carcinoma
HPV	Human papillomavirus
HNC	Head and Neck Cancer
HNSCC	Head and neck squamous carcinoma
HPV(+)	Human papillomavirus-positive
HPV(−)	Human papillomavirus-negative
WES	Whole-exome sequencing
SCC	squamous cell carcinoma
WGS	Whole-genome sequencing
CYLD	Cylindromatosis
EBERs	Epstein-Barr virus (EBV)-encoded small RNAs
LMP1	Latent-membrane protein 1
PDXs	Patient-derived xenografts
NSCLC	Non-small cell lung cancer
TRAF3	TNF receptor associated factor 3
RTK	receptor-tyrosine kinase
